# A Ketogenic Formula Prevents Tumor Progression and Cancer Cachexia by Attenuating Systemic Inflammation in Colon 26 Tumor-Bearing Mice

**DOI:** 10.3390/nu10020206

**Published:** 2018-02-14

**Authors:** Kentaro Nakamura, Hidekazu Tonouchi, Akina Sasayama, Kinya Ashida

**Affiliations:** Nutrition Research Department, Food Science & Technology Research Laboratories, R&D Division, Meiji Co., Ltd., 1-29-1 Nanakuni, Hachiouji, Tokyo 192-0919, Japan; hidekazu.tonouchi@meiji.com (H.T.); akina.sasayama@meiji.com (A.S.); kinya.ashida@meiji.com (K.A.)

**Keywords:** ketone body, ketogenic formula, colorectal cancer, cancer cachexia, inflammation

## Abstract

Low-carbohydrate, high-fat diets (ketogenic diets) might prevent tumor progression and could be used as supportive therapy; however, few studies have addressed the effect of such diets on colorectal cancer. An infant formula with a ketogenic composition (ketogenic formula; KF) is used to treat patients with refractory epilepsy. We investigated the effect of KF on cancer and cancer cachexia in colon tumor-bearing mice. Mice were randomized into normal (NR), tumor-bearing (TB), and ketogenic formula (KF) groups. Colon 26 cells were inoculated subcutaneously into TB and KF mice. The NR and TB groups received a standard diet, and the KF mice received KF *ad libitum*. KF mice preserved their body, muscle, and carcass weights. Tumor weight and plasma IL-6 levels were significantly lower in KF mice than in TB mice. In the KF group, energy intake was significantly higher than that in the other two groups. Blood ketone body concentrations in KF mice were significantly elevated, and there was a significant negative correlation between blood ketone body concentration and tumor weight. Therefore, KF may suppress the progression of cancer and the accompanying systemic inflammation without adverse effects on weight gain, or muscle mass, which might help to prevent cancer cachexia.

## 1. Introduction

Many cancer patients develop malnutrition, making them vulnerable to cachexia. Cancer cachexia is a complex metabolic disorder that occurs in association with cancer and characterized by loss of weight and skeletal muscle [1]. Cancer cachexia is a clinical issue because it not only causes weight loss, but also diminishes the quality of life (QOL) of cancer patients and has a negative effect on treatment compliance [2]. This condition continues to worsen while tumor progression is not suppressed. However, cancer treatments such as chemotherapy and radiotherapy might also have adverse effects on patient nutritional status [3]. Therefore, treatment should simultaneously maintain nutritional status while shrinking tumors. In recent years, multimodal therapy, combining drug, nutrition, and non-drug therapies for cachexia in addition to conventional therapy, have been developed, and studies have determined that this might be more effective than monotherapy for cancer treatment [4].

Cancer has recently been regarded as a metabolic disease, and studies have started to examine metabolic therapy using a ketogenic diets as a complementary or alternative therapy for cancer [5]. The ketogenic diet is a low-carbohydrate, high-fat diet designed to increase the blood concentration of ketone bodies as an alternative source of energy to glucose [6]. When humans experience insufficient glucose supply because of fasting or long-term exercise, they metabolize fat and generate ketone bodies (β-hydroxybutyrate and acetoacetate) from fatty acids as sources of energy [7]. The ketogenic diet has been prescribed since the 1920s to treat refractory epileptic seizures in children [8]. As the ketogenic diet switches the energy source for cells from glucose to fatty acids and ketone bodies, it is also used for the dietary management of specific metabolic disorders such as glucose transporter 1 (GLUT1) deficiency and pyruvate dehydrogenase complex deficiency [7]. In Japan, a special infant formula with a high-fat, low-carbohydrate composition, which is formulated with medium chain triglycerides (MCTs) with a ketogenic ratio of 3:1, has long been used for ketogenic dietary therapy for infants with congenital metabolic disorders and refractory epilepsy [9].

In most cancer cells, oxidative phosphorylation in the mitochondria is inadequate, even in the presence of oxygen, and energy from anaerobic glycolysis is enhanced to compensate for this (the Warburg effect) [10]. Cancer cells thus require large amounts of glucose. As cancer cell mitochondria are dysfunctional, they cannot readily use fatty acids and ketone bodies, which can be used as energy sources by healthy cells [10]. The Warburg effect is the target of ketogenic dietary therapy for cancer, as such diets aim to limit energy sources for cancer cells by restricting carbohydrates, while providing fatty acids and ketone bodies as an energy source for healthy cells [5].

The majority of previous studies on the anti-tumor effects of ketogenic diets have focused on specific types of cancer including brain, prostate, and breast, and few studies have addressed such effects on colorectal cancer [7,11,12]. In addition, it is also unknown if Ketonformula can be used for ketogenic dietary therapy to treat tumors. In this study, we therefore evaluated the anti-tumor and anti-cachexia effects of Ketonformula in tumor-bearing mice inoculated with colon 26 colorectal tumor cells.

## 2. Materials and Methods

### 2.1. Cell Lines and Culture

The mouse tumor cell line, colorectal adenocarcinoma colon 26, derived from BALB/c mice, was kindly provided by the Japanese Foundation for Cancer Research (Tokyo, Japan). Colon 26 tumor cells were maintained in RPMI 1640 medium with l-glutamine (Invitrogen, Tokyo, Japan) containing 10% fetal bovine serum.

### 2.2. Animals

Seven-week-old male CDF1 mice were purchased from Japan SLC (Hamamatsu, Japan). The mice were housed in plastic cages under controlled temperature and humidity with a 12-h light/dark cycle and fed commercial chow with water *ad libitum* for 1 week prior to use in experiments. The experiments reported herein were approved by the Animal Experiment Committee of Meiji Co., Ltd. (Tokyo, Japan) and carried out in strict accordance with the guidelines of this committee (approval #2013_3871_0079, approval date 26 June 2013). All efforts were made to minimize animal suffering, and when symptoms including severe body weight loss, hunching behavior, etc., were observed in a mouse, the mouse was euthanized by cervical dislocation.

### 2.3. Experimental Design

After acclimation, 25 mice were randomized into three groups: normal (NR; *n* = 5), tumor-bearing control (TB; *n* = 10), and ketogenic formula (KF; *n* = 10). Colon 26 cells (5.0 × 10^5^) in 0.1 mL phosphate-buffered saline were subcutaneously inoculated into the right flank of TB and KF mice on day 0. The NR and TB groups received the standard rodent diet, AIN-93G, and the KF group received the ketogenic formula, Ketonformula^®^ 817-B (Meiji Co., Ltd., Tokyo, Japan), *ad libitum* for 3 weeks. The compositions of the diets are listed in Table 1. Food intake and body weight were measured twice per week. Mice were euthanized on day 21 to collect their blood and tissues.

### 2.4. Measurement of Plasma Prostaglandin E_2_ (PGE_2_), Cytokine, and β-Hydroxybutyrate Levels

Plasma interleukin-6 levels were analyzed using a mouse cytometric bead array inflammation kit (BD Biosciences, San Diego, CA, USA) on a FACSCalibur flow cytometer affixed with a 488-nm laser (BD Immunocytometry Systems, San Jose, CA, USA), according to the manufacturer’s protocol. Plasma PGE_2_ levels were determined using commercially available purification kits and enzyme-linked immunosorbant assay kits (Cayman Chemical, Ann Arbor, MI, USA), according to the manufacturer’s protocols. Blood β-hydroxybutyrate (βHB) levels were measured enzymatically on day 21 using the Precision Xceed system (Abbott, Tokyo, Japan) to evaluate the production of ketone bodies and to investigate the correlation between ketone bodies and anti-tumor efficacy.

### 2.5. Statistical Analyses

Data are presented as means ± standard deviations. Differences among the three groups were assessed using the Bartlett’s test for variance, followed by the Tukey-Kramer test for homoscedastic data or the Steel-Dwass test. Comparisons between the TB and KF groups were performed using Student’s or Welch’s *t*-tests. The relationship between blood βHB levels and tumor weight was investigated by calculating Pearson’s correlation coefficients. Differences were considered significant at *p* < 0.05.

## 3. Results

### 3.1. Effect of Ketogenic Formula on Body, Tumor, and Tissue Weights

**Figure 1:**
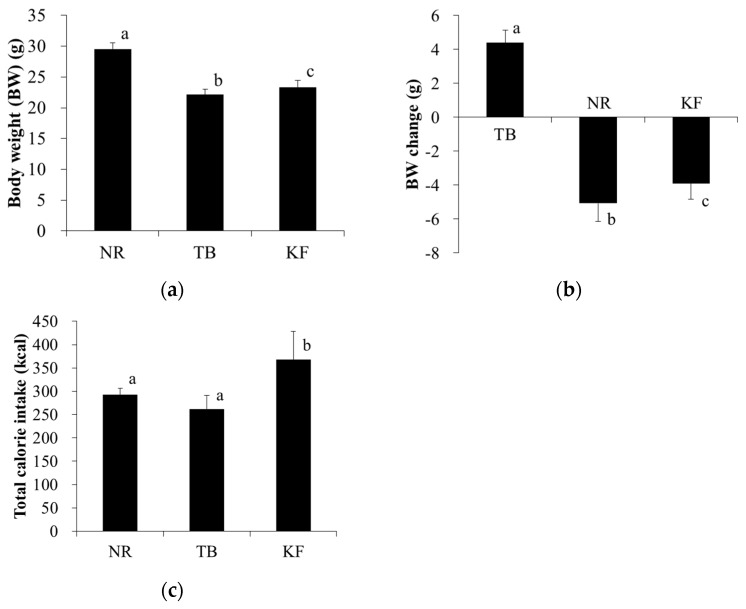
Effect of ketogenic formula on weight gain and total energy intake in mouse model of colorectal cancer. Legend: (**a**) Body weights and (**b**) body weight gain at the day of necropsy (day 21 after tumor inoculation); and (**c**) total energy intake. Values are means ± standard deviations, and values with different superscripts are significantly different (*p* < 0.05). NR, normal group; TB, tumor-bearing group; KF, ketogenic formula group.

Weight and weight change at day 21 were both significantly lower in the TB group than in the NR group (Figure 1a,b). Although weight and weight change were also both significantly lower in the KF group than in the NR group, they were significantly higher compared to that in the TB group (Figure 1a,b). Total energy intake during the experimental period was significantly higher in the KF group than in the other two groups (Figure 1c).

**Figure 2:**
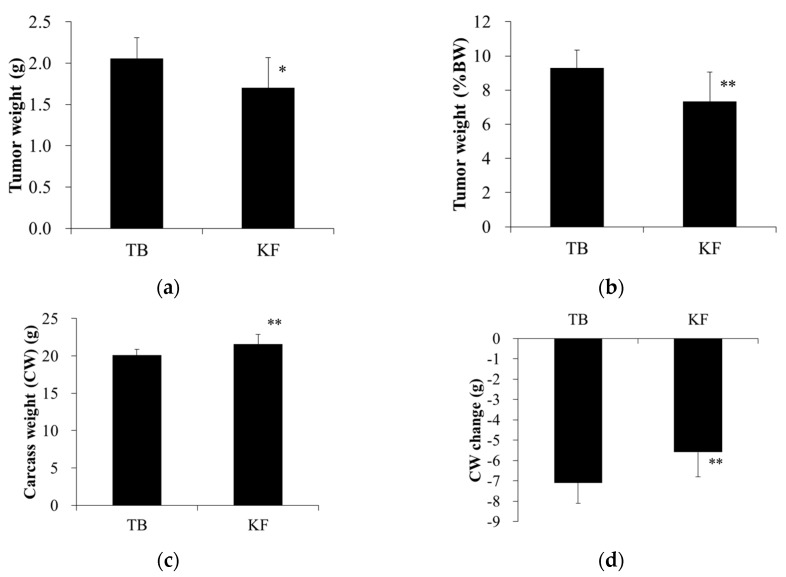
Effect of ketogenic diet on tumor weight and carcass weight in a mouse model of colorectal cancer. Legend: (**a**,**b**) tumor weights and (**c**,**d**) carcass weights at the day of necropsy (day 21 after tumor inoculation). Values are means ± standard deviations. * *p* < 0.05, ** *p* < 0.01, vs. TB group. BW, body weight; TB, tumor-bearing group; KF, ketogenic formula group.

Tumor weight was significantly lower in the KF group than in the TB group (Figure 2a,b). Similar to body weight, carcass weight and change in carcass weight were both significantly greater in the KF group than the TB group (Figure 2c,d).

**Figure 3:**
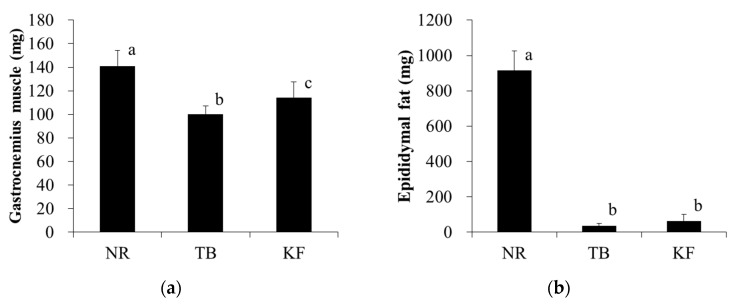
Effect of ketogenic diet on gastrocnemius muscle and epididymal fat mass in a mouse model of colorectal cancer. Legend: Tissue weights were recorded on the day of necropsy (day 21 after tumor inoculation). (**a**) gastrocnemius muscle; (**b**) epididymal fat. Values are means ± standard deviations, and values with different superscripts are significantly different (*p* < 0.05). NR, normal group; TB, tumor-bearing group; KF, ketogenic formula group.

In addition, gastrocnemius muscle mass was also significantly lower in the TB group than in the NR group; however, in the KF group it was significantly higher than that in the TB group (Figure 3a). Epididymal fat mass was significantly lower in both the TB and KF groups than in the NR group, with no significant difference between the KF and TB groups (Figure 3b).

### 3.2. Effect of Ketogenic Formula on Plasma Mediators of Inflammation

**Figure 4:**
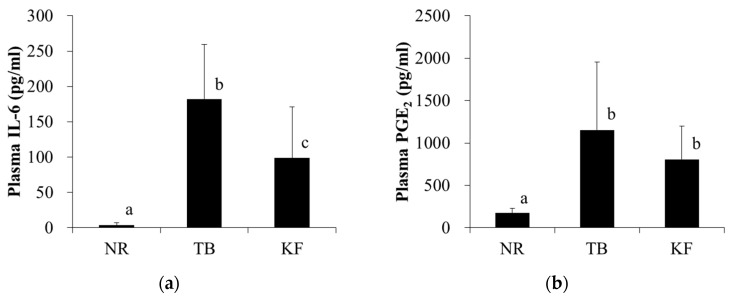
Effect of ketogenic diet on mediators of inflammation associated with colorectal tumors. Legend: Plasma levels of inflammatory mediators were assessed on the day of necropsy (day 21 after tumor inoculation). (**a**) interleukin-6; (**b**) prostaglandin E_2_. Values are means ± standard deviations, and values with different superscripts are significantly different (*p* < 0.05). NR, normal group; TB, tumor-bearing group; KF, ketogenic formula group.

Plasma IL-6 and PGE_2_ concentrations were significantly higher in the TB group than in the NR group (Figure 4a,b). Plasma IL-6 concentrations were significantly lower in the KF group than in the TB group; however, there was no difference in plasma PGE_2_ concentrations between the KF and TB groups (Figure 4a).

### 3.3. Correlation between Blood βHB Levels and Tumor Progression

**Figure 5:**
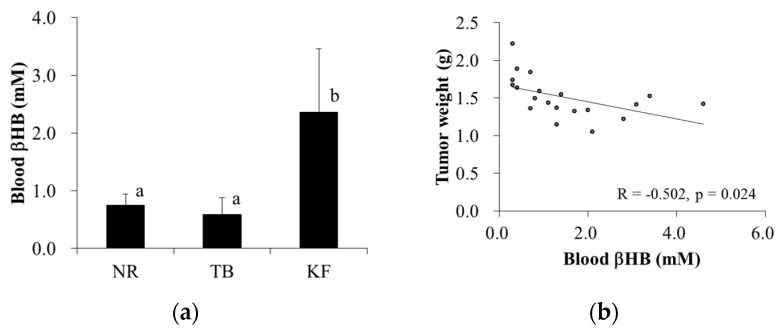
Effect of ketogenic diet on β-hydroxybutyrate (βHB) levels in a mouse model of colorectal cancer. Legend: (**a**) blood βHB levels were assessed on the day of necropsy (day 21 after tumor inoculation); (**b**) correlations between blood βHB levels and tumor weights were determined. Values are means ± standard deviations, and values with different superscripts are significantly different (*p* < 0.05).

Plasma βHB concentrations were approximately three-fold higher in the KF group than in the other two groups, and this was significantly different (Figure 5a). Moreover, there was a significant negative correlation between plasma βHB concentration and tumor weight in the TB and KF groups (*r* = −0.572, *p* = 0.024) (Figure 5b).

## 4. Discussion

In this study, we found that KF, which is used for children with congenital metabolic disorders or refractory epilepsy, suppressed the progression of murine colon 26 tumors, even in the absence of caloric restriction. KF also significantly suppressed the systemic inflammatory response and muscle loss associated with cancer progression. Most previous studies on the in vivo effect of a ketogenic diet on tumors have focused on brain, breast, or prostate cancers [7,11,12]. Few studies have addressed such effects on colorectal cancer, although one study reported that a low-carbohydrate diet had no anti-tumor effect [13]. A ketogenic diet has not been found to be effective in all laboratory animal models, and some studies have stated that caloric restriction is required to elicit the anti-tumor effects of such diets [11,14]. Ours is the first study to demonstrate the anti-tumor effect of a ketogenic diet on colon 26 cells without caloric restriction. Our study used a model of subcutaneous inoculation of colon cancer cells. Thus, future studies on the effect of a ketogenic diet on colorectal cancer and other cancers of the gastrointestinal tract could elucidate its effect on gastrointestinal cancer in greater detail.

In this study, adverse effects were not observed in mice fed with the KF. The KF in this study is the low level of the protein content (8% of energy), but that is common in ketogenic diets. We did not choose the diet with standard protein levels, because there is concern that the ketogenesis is not induced due to the influence of protein-derived gluconeogenesis, even though carbohydrates are restricted [15]. It has been reported that the lean body mass is not affected by the protein levels of the ketogenic diet [16], and we observed the same thing in the preliminary experiment (data not shown).

Our results suggest that tumor size might be correlated with blood βHB concentration, indicating that one mechanism for the anti-tumor effect of KF might be the elevation of βHB concentration as a result of KF intake. The systemic inflammatory response has been shown to be important for the development and progression of cancer and cancer cachexia [17,18]. βHB itself has also been reported to exert an anti-inflammatory effect [19,20], and to suppress IL-1β and IL-18 production via NLRP3 inflammasomes in human monocytes [20]. Moreover, βHB can also suppress mRNA expression of the inflammatory cytokines TNF-α, IL-1β, and IL-6 in the microglia of a rat model of lipopolysaccharide-induced Parkinson’s disease [19]. In our study, we demonstrated that the elevation of plasma IL-6 concentration was inhibited in the KF group, suggesting that βHB induction by KF ingestion might have suppressed the systemic inflammatory response and thereby inhibited cancer progression.

The results of previous clinical studies have also suggested that elevated blood βHB concentrations might have anti-tumor effects. In a clinical trial of a ketogenic diet for cancer patients, blood βHB concentration was three-fold higher in patients who achieved stable disease (SD) or partial remission (PR) compared to those with progressive disease (PD) [21]. According to Klement et al., tumor size decreased significantly when patients consumed a calorie-restricted ketogenic diet, whereas an *ad libitum* ketogenic diet had no anti-tumor effect [14]. Blood βHB concentrations in patients administered with a ketogenic diet without caloric restriction might not have risen sufficiently to exert an anti-tumor effect. Although that trial used a ketogenic diet with a high ketogenic ratio of 4:1, it did not use MCTs, which are associated with a high potential for ketone body production [22]. If a ketogenic diet formulated with MCTs had been used, it is possible that βHB production would have been sufficient to obtain an anti-tumor effect, even without caloric restriction. In one animal study utilizing a diet with a low ketogenic ratio (1.5:1), it was found that, despite using chow containing limited glucose and a high fat composition, a low-carbohydrate diet had no anti-tumor effect [13]. This relatively low ketogenic ratio diet might not have been sufficient to elevate blood βHB concentrations, resulting in a lack of anti-tumor effects. In light of the findings of these studies, the reasons as to why the KF produced an anti-tumor effect, even when the mice were fed *ad libitum*, might include the high ketogenic ratio of the diet and the fact that it is formulated with MCTs.

Nevertheless, it is possible that the observed anti-tumor effects of the KF might have been unrelated to blood βHB concentrations, with both tumor reduction and elevated blood βHB concentrations caused by glucose restriction, and the two factors exhibiting an indirect association. Tumor cells use different metabolic pathways compared to that in healthy cells, with the majority depending on glycolytic pathways and having limited ability to obtain energy from oxidative phosphorylation; hence, restriction of glucose can inhibit tumor growth [10]. Glucose restriction also increases ketone body production in the liver, elevating blood βHB concentrations [10]. The suppression of the systemic inflammatory response achieved in this study might also have been a secondary effect of the anti-tumor effect of the ketogenic diet, rather than a direct anti-inflammatory effect related to βHB. Further studies are required to elucidate this associated mechanism.

In this study, we found that dietary management with Ketonformula resulted in the successful maintenance of both body weight and muscle mass. Although some previous studies have found that cancer-associated weight loss was improved with a ketogenic diet, none reported an improvement in muscle mass [23,24]. The reason that both body weight and muscle mass were maintained by Ketonformula might be related to both the inhibition of cancer progression and the composition of the formula. In healthy tissue, ketone bodies can be taken up by cells and used to produce ATP via the mitochondria [6]. To increase physiological ketone body production, Ketonformula includes only a limited amount of carbohydrates; hence, although cancer cells might have had an insufficient supply of glucose, healthy tissues were maintained because they received energy from ketone bodies. Mice in the KF group exhibited higher energy intake than those in the other two groups, and this might have also been a factor in the maintenance of weight and muscle mass. In addition to its high-energy and high-fat composition, Ketonformula is formulated with MCTs, which might also have contributed to the increased energy intake. MCTs have been shown to increase levels of acyl-ghrelin, a peptide hormone that is primarily secreted by the stomach, and enhances growth hormone secretion and appetite [25,26,27]. For the activity of ghrelin, its third serine must be modified with octanoic acid, one of the fatty acids found in MCTs [27]. Indeed, feeding mice with chow containing MCT increases the concentration of acyl-ghrelin in the stomach [25]. In addition, when patients with chronic respiratory disease were given an enteral formula with a high MCT content, their plasma acyl-ghrelin concentrations, appetite scores, and weight all increased [26]. Ketonformula has a high MCT content, mainly octanoic acid, and it is possible that levels of activated ghrelin increased in mice in the KF group, elevating their energy intake. Moreover, colon 26 tumor-bearing mice are an animal model in which loss of appetite does not occur [28]. We also found no significant difference in energy intake between mice in the NR and TB groups, making it unlikely that the increased energy intake of mice in the KF group was due to its anti-tumor effect.

The anti-inflammatory effect of βHB might also have contributed to the maintenance of muscle mass and weight in the KF group. The muscle loss that occurs during cancer cachexia is a result of reduced synthesis and increased degradation of skeletal muscle [18]. IL-6 and other inflammatory cytokines promote muscle degradation by activating the ubiquitin-proteasome pathway. Such muscle degradation occurs via induction of the expression of the muscle-specific E3 ligase, Atrogin-1 (also called MAFbx), and muscle RING finger protein 1 (MuRF-1). Future studies on the effect of Ketonformula and βHB on Atrogin-1, MuRF-1, and associated cachexia are required to ascertain whether this hypothesis is correct. FGF21 is known as a major metabolic regulator of glucose and lipid homeostasis [29], and is involved in the metabolic benefits of the ketogenic diets [30]. Previous studies have demonstrated that FGF21 is not required for ketogenesis induced by ketogenic diets [31] and is independent of the tumor-suppressing effects of the ketogenic diet [30]. Therefore, FGF21 is thought not to be involved in the tumor-suppressing effects of the KF.

A number of clinical trials of ketogenic dietary therapy for cancer have been conducted and although evidence is still lacking, results suggest that this strategy might be effective and safe. A case report of a 65-year-old woman with glioblastoma multiforme who adopted a ketogenic diet for two months while also undergoing chemotherapy and radiotherapy reported that her blood ketone body concentration increased and the tumor disappeared [32]. A pilot study in which 16 patients with advanced metastatic cancer adopted a ketogenic diet for 3 months found that the outcome was PD for five of the patients who were unable to continue with the trial, whereas the outcome was SD for the five who completed the trial [33]. Those five patients who completed the trial also reported improvements in QOL scores (for emotional function) and insomnia, with no serious side effects other than constipation and fatigue, and with no deviations in blood cholesterol or triglyceride levels. In the ERGO trial of a ketogenic diet with 20 patients with recurrent brain tumors, the adoption of a ketogenic diet prolonged progression-free survival with no serious side effects attributable to the diet [34].

The use of Ketonformula in ketogenic dietary therapy has a number of advantages. The first is that Ketonformula has long been used for the treatment of patients with congenital metabolic disorders and epilepsy, and both, its capacity for ketone body generation and its safety for human consumption have both been demonstrated [9]. The second is that Ketonformula is a powder that does not contain liquid. As we could use it in place of flour, it could be used as the ingredient for a wide range of ketogenic meals. The third is that it also contains appropriate amounts of vitamins and minerals. Ketogenic dietary therapy has been reported to result in vitamin or mineral deficiency based on the fact that available foods for the therapy are limited [6,35]. Ketonformula might thus help to prevent such micronutrient deficiencies. Ketonformula might also result in some abdominal symptoms that are often caused by MCT ingestion (data not shown). These findings indicate that Ketonformula might be useful for ketogenic dietary therapy. Clinical trials are required to establish the efficacy and safety of ketogenic dietary therapy including Ketonformula for cancer patients.

Weight loss affects the outcome of cancer therapy, and is an important factor for the prognosis and QOL of cancer patients [36]. Dietary management using Ketonformula to maintain weight and muscle mass might contribute to improving the prognosis and QOL of cancer patients. One limitation of this study is that although we demonstrated the short-term anti-tumor and anti-inflammatory effects of Ketonformula, we did not evaluate long-term survival. Additionally, we did not investigate the effect of a ketogenic diet in combination with conventional chemotherapy and radiotherapy. Recent studies have found that fish oil and eicosapentaenoic acid (EPA), which are effective against cachexia, dampen the anti-tumor effect of cisplatin, and this should be considered when using fish oil or EPA during chemotherapy [37]. Further studies are required to investigate whether a ketogenic diet interferes with the anti-tumor effects of chemotherapy or radiotherapy or amplifies their side effects.

## 5. Conclusions

Dietary management with Ketonformula can suppress colon tumor progression and systemic inflammatory responses, and improving body and muscle weights, which might help to prevent cancer cachexia. This study also suggested the importance of cancer treatment based on metabolic changes induced by cancer load. Further studies on the use of Ketonformula for ketogenic dietary therapy in cancer patients might provide additional evidence of its value for the treatment of cancer cachexia.

## Figures and Tables

**Table 1:** Nutritional contents of the test diets (per 100 kcal).

	Ketogenic Formula	Rodent Purified Diet AIN-93G
Carbohydrates (g)	1.2	16.1
Proteins (g)	2.0	5.0
Lipids (g)	9.7	1.7
MCT ^1^ (g)	5.4	0.0
Others	Vitamin mix, mineral mix	Vitamin mix, mineral mix
Ketogenic ratio ^2^	3.0	0.08
Carbohydrates (% kcal)	5	64
Proteins (% kcal)	8	20
Lipids (% kcal)	87	16

^1^ MCT, medium chain triglycerides. ^2^ Ketogenic ratio, the ratio of the weight of lipids to carbohydrates and proteins.
